# Transforming Shame in the Pandemic: An International Study

**DOI:** 10.3389/fpsyg.2021.641076

**Published:** 2021-06-14

**Authors:** Claude-Hélène Mayer, Elisabeth Vanderheiden

**Affiliations:** ^1^Department of Industrial Psychology and People Management, University of Johannesburg, Johannesburg, South Africa; ^2^Kulturwissenschaftliche Fakultät, Europa Universität Viadrina, Frankfurt, Germany; ^3^Global Institute for Transcultural Research, Römerberg, Germany

**Keywords:** shame, transforming shame, COVID-19, thematic analysis, positive psychology 2.0, meaning, mental health

## Abstract

Shame is an unconscious, somehow unattended and neglected emotion and occurs when individual and socio-cultural norms are violated. It often impacts negatively on the self and others across cultures. During the Covid-19 crises, shame has become an important emotion with a powerful effect, depending on how it is experienced within the socio-cultural context. This article explores shame in international perspectives in the context of Covid-19 and addresses the question how shame is transformed from an existential positive psychology (PP2.0) perspective. The study uses a qualitative research paradigm and explores shame and its transformation during Covid-19. Purposeful and snowball sampling was used. The sample consisted of 24 individuals (16 female, 8 male), of 13 different nationalities. Data were collected from written interviews and analyzed through thematic analysis. Ethical considerations were followed; ethical approval was given by a university. Findings show that participants become very worried, anxious, scared, sad, and shocked when they or individuals in their close relationships contracted Covid-19. Shame plays an important role during the Covid-19 pandemic. However, the meaning and experience of shame during Covid-19 is strongly dependent on the socio-cultural background of the individual who is experiencing the disease. Individuals use different strategies and mechanisms to deal with and transform shame in the context of Covid-19.

## Introduction

The new coronavirus disease (COVID-19) has put the world into a pandemic and a (mental) health crisis (World Health Organization (WHO), 2020). The spread of the virus has caused havoc in the personal and professional lives of individuals, through losses and unemployment (International Labor Organization (ILO), 2020). This outbreak does not only cause not only material losses, but also impacts on the mental health and well-being of individuals due to social distancing, home containment and lockdown situations, travel restrictions, or other mitigation strategies (Restubog et al., 2020). Individuals in quarantine feel stigmatized, ostracized and rejected (Bai et al., 2004) and experiences increase the emotional strain (Sonnentag et al., 2010; Kossek et al., 2012) which needs to be regulated or transformed (Restubog et al., 2020; Sun et al., 2020).

Shame as an extraordinary, negative emotion has gained interest in research. It can be transformed into a resource and become a coping mechanism (Mayer and Vanderheiden, 2019; Mayer et al., 2021). A PP2.0 perspective aims at transforming negative emotions and shame, through compassion, empathy and the increased awareness of the frailty and vulnerability of all humans (Wong, 2017).

Since 2011 existential positive psychology (PP2.0) has gained in importance, dealing with the questions how to lead a meaningful and a positive and constructive life at the same time (Wong, 2011; Passmore and Howell, 2014; Ivtzan et al., 2017; Carreno and Pérez-Escobar, 2019). Therefore, research in PP2.0 suggests that the dark and negative aspects of life, the suffering and the pain of existence need to be radically accepted, dealt with and transformed—if this is the case, mental health and well-being improves based on the lived-through experience (Wong, 2011, 2016, 2020; Fowers et al., 2017; Turaniu et al., 2017; Vanderheiden and Mayer, 2017; Mayer and Vanderheiden, 2019; Van Tongeren and Showalter Van Tongeren, 2020).

The aim of the article is to present the voices of international individuals who experience shame during Covid-19. The article thereby contributes to the multidisciplinary and multicultural PP2.0 view on shame to deal with it constructively and as a health resource (Vanderheiden and Mayer, 2017; Mayer and Vanderheiden, 2019; Mayer, 2020; Mayer et al., 2021). The key question in the study is: *How is shame experienced during Covid-19 and how can it, if necessary, be transformed?*

## Existential Positive Psychology (PP2.0) to Transform Shame

What PP2.0 entails and how it developed from the first positive psychology movement (PP1.0) has been previously discussed and described extensively (Wong et al., 2021). For the present article, selected aspects of the PP2.0 and shame and its transformation are described briefly as forming the theoretical base of PP2.0. PP2.0, dealing with existential questions and a positive view on challenges in life, can support individuals through suffering, pain, mass anxiety, fear of death, sickness, and human weaknesses (Wong, 2020). The conscious recognition of the suffering caused by, for example, a negative emotion such as shame, needs to be radically accepted and later on transformed into deeper meaningfulness, existential joy and happiness (Wong, 2020).

Well-being can then be achieved based on meaningful experiences, joy and happiness by transforming dark and negative emotions, such as shame (Vanderheiden and Mayer, 2017; Mayer and Vanderheiden, 2019; Mayer et al., 2021).

**Figure 1:**
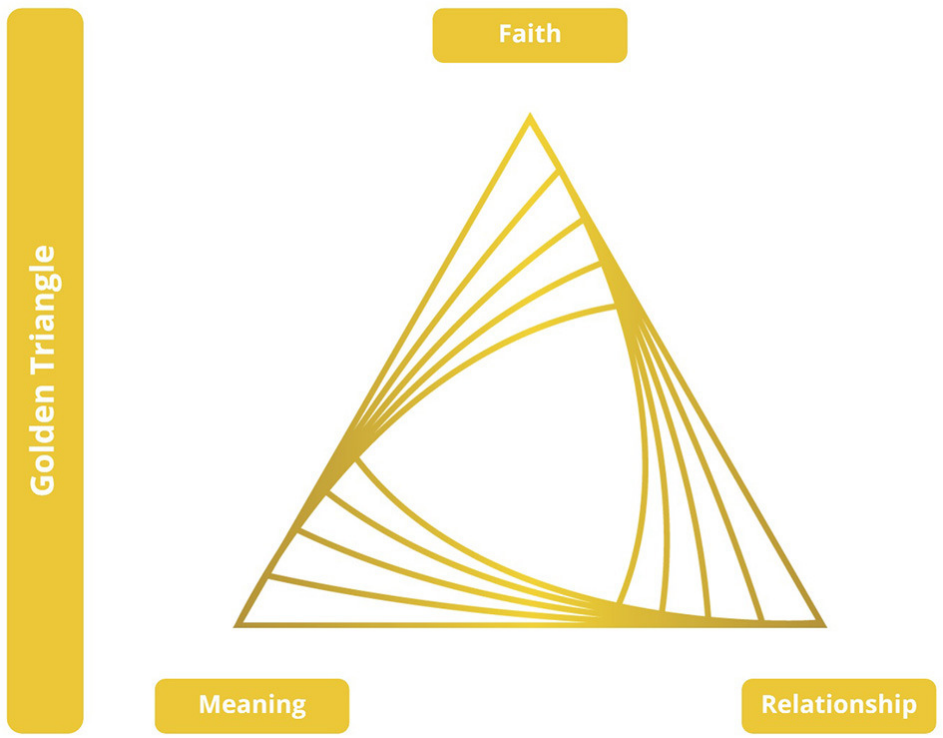
The Golden Triangle, based on Wong (2019).

Wong (2019) has pointed out that the golden triangle for positive mental health consists of faith (in a transcendental reality and intrinsic value of life), meaning (life goal or value more important than self), and relationship (mutual trust and care) (Figure 1).

**Figure 2:**
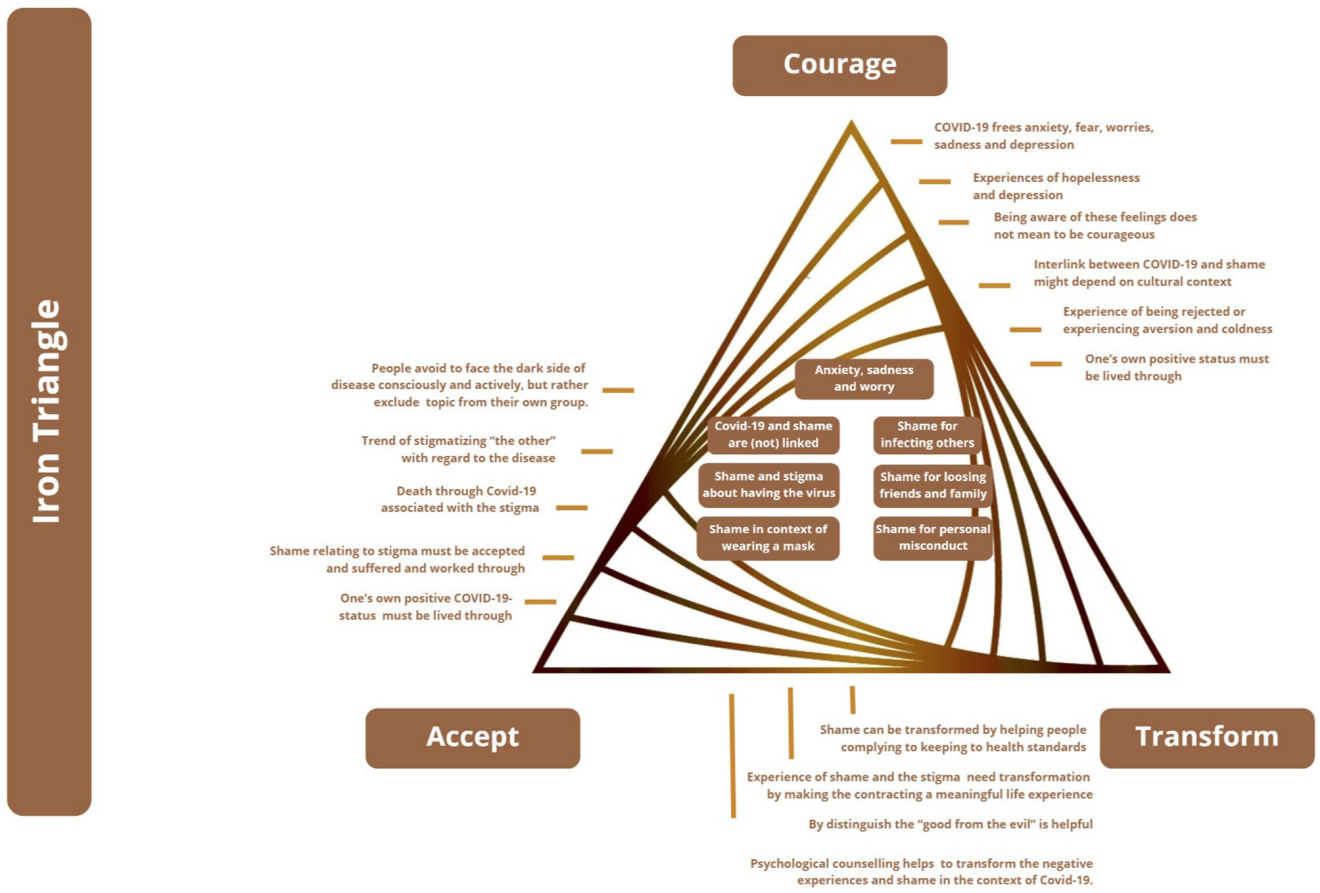
The Iron Triangle, based on Wong (2019).

In parallel to the golden triangle for positive mental health, the iron triangle (Figure 2) deals with the terrors of life, and is based on courage (to face the dark side of life), acceptance (what cannot be avoided or changed), and transformation (suffering into strength through meaning).

PP2.0's mission is to provide responses on how to transform the iron triangle into the golden triangle. The acceptance of pain, despair, and suffering is viewed as integral to the process of enhancing happiness, not as a separate process (Wong, 2011; Wong and Worth, 2017). Frankl (1959) explain how a huge personal (or even societal) life crisis can be turned into a positive resource when individuals tap into the courage to suffer. Through pain and suffering, individuals can finally find meaning and existential resilience and through addressing shame, a transformation from negative toward positive shame can take place.

## Shame in International Perspectives During COVID-19

Emotions and their impact on well-being in the pandemic are currently being researched (Giallonardo et al., 2020; Parlapani et al., 2020; Rossi et al., 2020). The pandemic appears to have a reinforcing effect on existing mental disorder symptoms (Torales et al., 2020b). Concerning the role of shame, embarrassment, and humiliation in a pandemic, only a limited amount of research can be found (Haller et al., 2020; Sahoo et al., 2020; Siddiqui and Nowshin, 2020). However, none of these studies refers to shame as a potential resource, as understood in the context of PP2.0.

From an evolutionary perspective, shame can be described as a social regulator (Gilbert, 2019) that controls the relationship between proximity and distance, intimacy and publicity, belonging and demarcation, adaptation and autonomy. Scheff (2020) describes shame as a primary social emotion and sketches it as “the glue that holds relationships and societies together.” Wong (2017) describes shame as an existential emotion, common to all human beings. It is a “universal emotion” (Casimir and Schnegg, 2002; Sinha, 2017) but experienced in culture-relative ways (Vanderheiden and Mayer, 2017).

Shame can be felt by individuals, groups or entire societies and can be projected on them (Rushdie, 1989; Scobel, 2017). Shame manifests itself as culture-specific in terms of perception, experience, and expression (Miller, 1996; Lindisfarne, 1998) and is influenced by factors such as religion and the *Zeitgeist* (Greiner, 2014).

Walsh (2020) assumes that the experience of one's own vulnerability and dependence on others in the pandemic context, but also the experience of illness and death of others, can trigger strong emotions such as shame. In the Chinese cultural context, many Covid-19 patients suffer from feelings of shame and guilt, which can lead to depression (Duan et al., 2020). Others are ashamed since the suffer from food shortages (Weissman et al., 2020). In the Vietnamese context, people who accidentally spread the virus are shamed not only by individuals, but also by the government through newspaper and online shaming in combination with fake news (Max, 2020). Similar shaming processes for Covid-19 infected individuals could be recognized in Taiwan (Farr, 2020). In the Indian context, Sahoo et al. (2020) have described people's shame who have infected family members and professionals and their fear of shame and social exclusion. Kato et al. (2020) report that in Japan, potential Covid-19 sufferers are concerned about positive test results so that their status must remain unknown in their community. The authors see a close connection to traditional culture-based shame (*Haji*) and ostracism (*Murahachibu*) which, during past epidemics and economic crises, has often led to sick or financially ruined individuals committing suicide (Kato et al., 2020). Others report shame as an expression of stigmatization in the context of the Japanese context during the pandemic (Shigemura and Kurosawa, 2020, p. 1): healthcare workers were described as “germs” and publicly stigmatized. In case they worked with infected patients, they were expected to apologize publicly to those around them through a *Moushiwake Arimasen*, a culture-bound expression of apology and shame. The social stigmatization of Covid-19 sufferers and healthcare professionals has been reported repeatedly (Grover et al., 2020; Kahambing and Edilo, 2020; Singh and Subedi, 2020). In Bangladesh, Siddiqui and Nowshin (2020) found a close connection between shame and stigma in affected individuals: on the one hand, one infected man as the carrier of the disease was shamed as a spreader, while on the other hand, low-income women working in the garment industry were accused, stigmatized, and ashamed as super spreaders (Siddiqui and Nowshin, 2020, p. 4).

Le Brocq et al. (2020) surveyed overweight people in the United Kingdom who feared that they would not receive medical support if hospitalized, and were therefore at greater risk of dying. They felt stigmatized by the media. The study showed that those affected react to the stigma with shame; the increased risk of infection is expressed in increased body shame, and the lockdown itself also triggered fear of further weight gain. The situation increase fear, stigma, and shame, which prevented them from exercising or from shopping for food in a confident and self-assured manner (Le Brocq et al., 2020).

Gender differences in managing shame in the context of Covid-19 apply (Torales et al., 2020a) and researchers identify a worldwide increase in violence against women and children in connection with the panic lockdown. The victims keep silent about the violence due to shame while the perpetrators experience increased shame and torment (Bradbury-Jones and Isham, 2020). Lockdown and social isolation exponentially increase the risk of injury for women, shame and increasing violence in South Africa and North America (Buttell and Ferreira, 2020).

Katafuchi et al. (2020) point to a new Japanese social phenomenon in this context, the emergence of the *jishuku keisatsu* (“self-restraint police”). Members of the public see themselves as private police to pressurize individuals who not refrain from calling, going out, or other activities. Also in Switzerland shame is a valuable resource for young adults (15–20 years) to comply with public health measures (Nivette et al., 2021).

## Research Methodology

This study's research is based on a qualitative research design and uses a hermeneutic-phenomenological approach (Creswell, 2013). It is chosen to explore and understand the subjective experiences of shame and its transformation in the participants during Covid-19 (Hassan and Ghauri, 2014; Clarke and Hoggett, 2019).

### Sample

The researchers used two sampling techniques: purposeful sampling and snowball sampling (Palinkas et al., 2015). They approached individuals whom they knew, who had experienced Covid-19 themselves or had contact with individuals who have or had contracted Covid-19. The sample consists of 24 individuals (Table 1): 16 female and eight male participants. The age of the sample ranged from 22 to 81 years. Five individuals were between 20 and 30 years, four were between 31 and 40 years, five between 41 and 50, six between 51 and 60, two between 71 and 80, and one between 81 and 90. The participants hold 13 different nationalities; Germany (six participants), South Africa (four participants), the U.S. (three participants), Portugal (two participants). One participant came from each of the following countries: Canada, Australia, China, Argentina, the United Kingdom, Nigeria, Japan, India, and Romania. Table 1 provides insights into the biographical data and shows in which way the participants were affected by Covid-19.

**Table 1:** Biographical data.

**Participant**	**Age**	**Sex**	**Nationality**	**Country of residence**	**P affected by Covid-19 her/himself**	**P affected by Covid-19 by her/his partner**	**P affected by Covid-19 by a member of the family**	**P affected by Covid-19 by a friend**	**P affected by Covid-19 by a colleague**
P1	54	F	German	Germany	x				
P2	56	M	German	Germany		x	x		x
P3	22	F	German	Germany	x				
P4	24	F	German	Italian	x		x		
P5	45	F	German	South Africa				x	x
P6	82	M	United States	United States			x		
P7	54	M	Portuguese	Portugal	x	x	x	x	x
P8	48	F	Canadian	Canada	x		x		
P9	35	F	German	Germany			x		
P10	40	F	United States	United States			x		
P11	55	F	Australian	Australia				x	
P12	30	F	Chinese	Netherlands			x	x	
P13	68	M	British	United Kingdom	x				
P14	71	F	German/ Argentinierin	Germany				x	
P15	45	F	Portuguese	Portugal	x				
P16	51	M	Nigerian	Nigeria	x			x	
P17	23	F	South African	South Africa				x	
P18	58	F	United States	Germany			x	x	
P19	36	M	Japanese	United Kingdom					x
P20	24	M	South African	South Africa	x	x	x	x	x
P21	65	F	South African	South Africa				x	
P22	49	M	South African	South Africa					x
P23	40	F	Indian	India				x	
P24	45	F	Romanian	South Africa					x
					9	3	10	11	7

Selection criteria for participants were: being a minimum age of 18 years, having contracted the virus themselves or having experienced the contraction of the virus by individuals in their social environment such as family, friends, or colleagues, and finally being willing to participate in the study.

### Data Collection, Analysis, and Reporting

The researchers conducted written interviews by using a questionnaire containing the following questions with regard to biographical data, and the experience of shame and its transformation in the context of Covid-19:

- Biographical data: age, sex, national belonging, place of living, first language, contracted Covid-19 (self/others).
- How did you feel when you contracted Covid-19/ a person in a close relationship contracted Covid-19?
- Which role does/did shame play in the context of Covid-19?
- How do you/did you deal with shame in the context of Covid-19?
- Which resources support(ed) you in overcoming shame in the context of Covid-19?
- Which coping strategies do/did you use to overcome/transform shame?
- How does your society generally deal with Covid-19 and shame?

All written interviews were captured and analyzed through thematic analysis (Braun and Clarke, 2012). Thematic analysis is defined as “a method for systematically identifying, organizing, and offering insight into patterns of meaning (themes) across a data set” (Braun and Clarke, 2012, p. 57). The researchers used an abductive approach (Mayer, 2011), including the deductive and inductive thematic analysis method. This abductive approach includes the use of a template with codes having been developed prior to the coding of the transcribed interviews, while also allowing themes to emerge from the data (Fereday and Muir-Cochrane, 2006). According to Braun and Clarke (2012), thematic analysis consists of familiarization with the information gathered, which in this case involved reading and rereading the transcribed interviews. Thereafter codes were assigned and derived from the interview texts (Braun and Clarke, 2012) (Tables 2–5). The analysis of data was conducted through five steps as described by Clarke and Hoggett (2009): (1) the data was subjected to an initial, preliminary and holistic assessment, (2) themes were generated, (3) data was coded, (4) the body of the text was broken down into meaningful pieces which were labeled, and (5) closer attention was paid to the subtleties and nuances of the meaning inherent in the data by the researchers (Clarke and Hoggett, 2009). The researchers used inter-subjective validation processes, reflected upon and discussed the findings (Yin, 2014).

**Table 2:** Feelings when experiencing COVID-19.

	**All**	**Participants**
Worried/afraid/scared	13	P2, P4, P5, P6, P8, P9, P10, P11, P12, P13, P16, P17, P18
Sad	6	P5, P8, P9, P10, P12, P18
Shocked	3	P12, P14, P19
Felt sorry for someone else/concerned	3	P6, P17, P20
No answer	3	P15, P21, P24
Felt like always	1	P3
Calm	1	P1
Surprised	1	P2
Unsafe	1	P7
Exposed	1	P13
Hopeless	1	P10
Angry	1	P18
Exposed	1	P13
Guilty	1	P4
Hurt by other's people behavior	1	P13
Person affected was not close enough to affect me significantly.	1	P24
	39	

**Table 3:** The role of shame.

	**All**	**Participants**
No link between Covid-19 and shame	8	P1, P2, P3, P12, P13, P21, P23, P24
Shame helped individuals to wear masks	6	P7, P10, P15, P18, P19, P20
Shame due to contraction of the virus	5	P8, P14, P16, P17, P24
Shame for infecting others	2	P4, P9
Shame for personal misconduct	2	P6, P16
Shame over losing friends and family	1	P5,
Behavior of the government is shameful	1	P11
No answer	1	P22
	26	

**Table 4:** Strategies, resources, and coping to transform shame.

	**All**	**Participants**
No resources needed	11	P1, P3, P6, P9, P10, P11, P12, P13, P18, P21, P24
Social support and open talks	8	P2, P4, P14, P15, P16, P17, P18, P22
Spirituality, faith, and mindfulness	6	P4, P5, P7, P8, P19, P20, P22
Psychological counseling	2	P16, P23
Online resources	2	P20, P23
Informing oneself about the latest news, trends and information	2	P4, P15
Adhering to the rules and regulations of the government	2	P14, P20
	33	

**Table 5:** The societal impact on Covid-19 experiences.

	**All**	**Participants**
Exclusion, discrimination of specific ethnic groups	7	P2, P4, P5, P9, P10, P11, P21
Denial and avoidance and stigmatization	8	P1, P12, P13, P15, P16, P19, P22, P24
Too little or no connection between Covid-19 and shame	11	P3, P6, P7, P8, P9, P13, P14, P17, P18, P20, P23
Various reactions in society to deal with Covid-19 and shame possible	1	P22
	27	

The datasets were filed and are stored electronically for a period of 5 years. Data was reported using a qualitative reporting style, presenting qualitative and in-depth findings (Guba and Lincoln, 1994).

### Quality Criteria, Ethical Considerations, and Limitations

This qualitative study used quality criteria, as explained in the following: The individuals shared rich and detailed subjective experiences in their questionnaires. The detailed responses led to rigor in the quality of the data, its analysis and interpretation. Credibility was ensured through the triangulation of references, methods, and theories (Creswell and Plano Clark, 2011). Confirmability and transferability of data (Creswell, 2013) was promoted through intersubjective validation processes of the researchers and the use of established theories and methods chosen by the researchers (Yin, 2003, 2014). Rigor was promoted through transparent research processes and transcriptions, as well as the analysis of detailed and thick descriptions.

The study was conducted in an ethical manner, protecting the rights of the interviewees, anonymity and confidentiality (Taylor and Land, 2014). All participants were informed about confidentiality and their rights as participants. The study was approved by the German University. The participants provided individual consent to participate in the research.

The study is limited to a qualitative research approach in which participants were invited to provide information and describe their experiences of shame during times of Covid-19. The study included 24 participants with twice as many female participants as males and might therefore be affected by a gender bias. Six out of the 24 individuals hold German nationality, which might also affect the cultural bias of the findings. The study finally provides an in-depth insight into the findings and topics, but does not provide generalizability (Lincoln and Guba, 1985; Creswell, 2013).

## Findings

The findings are presented in order of the above research questions.

### Experiencing Covid-19

How did you feel when you contracted Covid-19/ a person in a close relationship contracted Covid-19?

Altogether, 13 participants highlighted that they were worried, afraid and scared when having contracted Covid-19 or when a close friend, relative or colleague was affected. Further, six participants felt “sad,” three each felt “shocked,” “sorry and concerned,” and three did not provide any answer.

A South African 45-year-old woman (P5) highlighted how, after her friend and colleague contracted Covid-19, she “felt sad and scared, anxious, and I was worried about the friend and the colleague.” A 48-year-old Canadian women emphasized: “I was scared, withdrawn and sad,” while another 23-year-old South African Afrikaans-speaking female participant said, “I was worried that I might have contracted Covid from my friend and I might have given it to an essential worker” (P17).

P4, a German 24-year-old female participant living and studying in Italy, was very worried because she contracted Covid-19 in Italy and brought it back to her family living in Germany: “I live in Italy. I study there. During the first lockdown I left Italy to see my family in Germany before the borders were closed. I returned to my family in Germany, so I brought the virus to them, unknowingly. I felt very guilty.”

One participant felt “exposed.” One person each emphasized that they felt calm, surprised, unsafe, hopeless, angry, guilty, hurt (by other people's behavior), or not affected. A 68-year-old man from the United Kingdom described the experience of having been in contact with people who had the virus and who did not protect him from being exposed to it: “I was exposed to others who were ill and even incubating the virus. This could have been avoided and wasn't. So I felt hurt by their behavior.”

The participants experienced a broad range of feelings, most of which were experienced as negative, such as worry, anxiety, concern, and sadness when being in close contact with the virus infections in themselves or in close others. Individuals felt a strong sense of guilt when they infected others, while participants who get infected by others owing to irresponsible behavior felt hurt and angry. Some of the participants highlighted that they do not feel affected by the contamination of individuals in their environment, as long as they do not have close relationships with them. They further explained that they feel surprised and shocked about close relationships contracting the virus, showing that they did not expect the direct impact of the virus in their close environment. One German 22-year-old female participant said that she felt as she always feels when she is sick; it was nothing special for her, while a 65-year-old South African was unable to express how she felt since a friend of hers passed away. Further, the description of sadness is often combined with feelings of fear, unsafety and hopelessness across cultures and is directly expressed by participants from China, South Africa, Germany, Portugal, and Canada. Women and men express similar feelings and no gender-specific differences revealed in the findings.

### The Role of Shame During Covid-19

Individuals were asked how Covid-19 and shame are interlinked.

#### Covid-19 Interlinkages With Shame

Altogether eight out of 24 participants do not see any link between Covid-19 and shame. Three people highlighted that shame plays a role in particular with regard to individual's behaviors since shame makes individuals feel humiliated and keeps them away from seeking help.

#### Shame in the Context of Wearing a Mask

Six participants emphasized that shame helped individuals to wear masks. Not wearing a mask in public when it is a societal requirement and norm makes people feel ashamed. Several participants from different cultural backgrounds highlighted both sides of shame, the negative and the positive: A male Portuguese 54-year-old participant (P7) said: “*Shame starts out being bad, because it brings us bad feelings. Then, it becomes something good if it corresponds to a change in our attitudes*.”

P19, a male Japanese participant aged 36 years and living in the United Kingdom, commented that shame is a positive force when it makes people wear a mask: “*In Japan, if you are not wearing a mask, you will be pointed to by strangers. Shame is part of emotions that are helping people wear a mask*.” A South African 24-year-old male participant (P20) agreed with regard to the positive effect of shaming and mask-wearing:

“Covid-19 changed the way we do things; we are unable to do lots of things now because of it and it is very painful to see that happening. To me, shame refers to a painful feeling of humiliation. So, some of the things we sometimes do, like not wearing a mask in public, could be shameful.”

Several participants highlighted shame's positive effect in terms of compliance to Covid-19 rules and regulations: Shame can transform from being a negative feeling to becoming a “life-saver” when wearing masks in public.

#### Shame and Stigma About Having the Virus

Five participants felt ashamed because they had contracted the virus. P8, a 48-year-old Canadian female participant told her story:

“I hear of people in other countries shaming people for either wearing or not wearing a mask. When I was just becoming sick, my ex-husband became very angry at me because he perceived that I had acted irresponsibly. I disagreed, but felt shamed in the conversation. Some friends reacted to hearing about my illness with aversion and coldness. Partly because of shame, I stopped telling people what was going on with me and forbade some others from sharing my news.”

Individuals are ashamed of having Covid-19 since it is associated with irresponsible behavior. The aversion and coldness as described contributes to a doubled shame experience: firstly based on the contracting of Covid-19, secondly based on the exclusion and isolation experienced.

P14, a 71-year-old female from Germany, reflected on the fact that being infected and feeling ashamed may well lead to not going to the doctor. This idea is supported by a male Nigerian participant aged 51 (P16): “Covid-19 brings stigma in Nigeria. It is not good as it does not allow people to seek help and treatment and those who have it continue to spread it due to the stigma surrounding it.”

A 45-year-old Romanian female (P24) living in South Africa pointed out:

“I assume some people who got infected felt ashamed for allowing that to happen to them? I would assume some Chinese nationals might feel ashamed because the whole thing started in their country. I am not sure how to answer this. Shame is usually a bad thing because it isolates people and make them depressed. I would say shame is good to reveal evil and can become a propelling force toward change.”

Overall, participants observed that the stigma of Covid-19 evokes or increases shame. Participants are aware that shame in the context of Covid-19 has positive, as well as negative functions: it might help to contain the virus, but it might also lead to negative mental health effects and rejection to see a doctor and to talk freely about the disease.

#### Shame for Infecting Others

Two participants felt ashamed because they had infected others with the virus. One German female, 24-year-old participant P4 said: “My mother has a serious pre-existing condition. She is a risk patient. I was ashamed of the fact that I infected and endangered her.”

When infecting others with the virus, the shame is particularly high since endangering others with the virus can lead to suffering, severe illness and death. In this context, shame was mentioned by two Germans who felt ashamed to having decreased the health of others and put their life at risk.

#### Shame for Personal Misconduct

Two participants felt ashamed because of the misconduct of people in their environment who did not adhere to the socially accepted conduct, such as P6, an 82-year-old male US-participant:

“My shame issues arise mainly from the actions of a first cousin who is important in my life, though she makes terrible decisions. She lives in Chicago, but was visiting her best friend in Florida when they both became infected with Covid-19. The friend was dying, but my cousin decided she could not help or be with the friend, so she flew home to Chicago, probably infecting people at two airports, people on her airplane, and her cab drivers. She lives in an enormous apartment building, and she went up and down in the building elevator while quite sick, to get to her apartment, to go for testing, to return from testing. I think she may have infected many people. If I were she, I would feel shame for abandoning my dying friend and for possibly infecting many people. She doesn't feel that way. For now, our different feelings about her actions makes me more distant from her. Maybe I also feel shame for judging her. She was doing what seemed best to her at the time.”

This individual feels ashamed for others who do not adhere to the social norm and health regulations released by the authorities or the government. He feels that they behave irresponsible.

#### Shame Over Losing Friends and Family

One participant felt ashamed because they had lost a friend or family member due to Covid-19. P5, a 45-year-old German, highlighted that she was not feeling any shame with regard to Covid-19, but she had seen it in a friend. “Not much for me, but for my friend who lost family, shame was a big issue.” A family might feel ashamed of the death of a family member, since contracting Covid-19 is associated with irresponsible behavior, personal (physical) weakness, ineffective treatment or care, and unaffordable healthcare.

Finally, one Australian female participant, aged 55 (P11), pointed out that the behavior of the government is shameful in connection with Covid-19. However, she did not provide a more in-depth explanation of this statement.

### Strategies, Resources, and Coping to Transform Shame

How do you/did you deal with shame in the context of Covid-19?

#### No Resources Needed

Altogether, 11 participants out of 24 stated that they did not have experience with shame in the context of Covid-19 and therefore did not need to activate any strategies or resources to overcome shame.

#### Social Support and Open Talks

Eight participants emphasized that they used social support as strategy to overcome shame and the virus. This social support can come in different forms; however, a majority of the eight individuals mentioned “open talks” with friends, colleagues, family, and spouses. This was especially important for female and male participants in Germany and Portugal, and for one male participant from the U.S. P2, a 56-year-old male participant from Germany explained: “What helps to overcome shame and Covid-19 is to talk about it. Talk openly.” Social support, expressing individual opinions, exchanging knowledge about the virus, and expressing feelings are indicators of a good relationship which is a key aspect of transforming shame. The open talk about Covid-19 is mainly mentioned by Western individuals.

#### Spirituality, Faith, and Mindfulness

Seven individuals said that they were supported by their spirituality and their faith to overcome shame in the context of Covid-19. P5, a female, 45-year-old German female referred to faith as an important resource in her life: “FAITH, to believe in myself, inner strengths, belief in a higher power, belief in humane and positive meaning of everything that happens in my life. That is important to overcome and transform shame.”

Particularly the African participants highlighted that faith and prayer are extremely important as resources to overcome shame. A Nigerian 51-year-old male (P16) noted: “Prayer and faith in God, encouraging people that it was not a death sentence to have Covid-19. That is important.”

#### Other Resources: Counseling and Information

Other resources used to overcome shame and Covid-19—each being mentioned twice—included: psychological counseling, online resources, informing oneself about the latest news, trends and information, and adhering to the rules and regulations of the government.

### The Societal Impact on Covid-19 Experiences

How does the society deal with Covid-19 and shame? While a male, 49-year-old South African (P22) explained that, P13, there were three overall trends indicated how societies reacted to Covid-19: (1) exclusion and discrimination, (2) denial and avoidance, and (3) too little or no connection of Covid-19 with shame.

#### Exclusion, Discrimination of Specific Ethnic Groups

Several individuals explained that in relation to Covid-19, many people see exclusion and discrimination happen within society. One German 24-year-old female participant (P4) explained:

“Here in Germany shame is something negative. And with regard to Covid-19, there is a lot of shaming going on with regard to members of certain ethic and social groups. Many are convinced that Covid-19 has been brought into the country by migrants and immigrants and that people with lower socio-economic background or standard get Covid-19 more easily. That is certainly nonsense and ridiculous.”

P9, a German female, 35 years old, described the situation in her region of Germany as follows: “This illness shames the people who contracted it. Near the city of G. in south-west Germany, for example, people with specific number plates on their cars, where Covid-19 is high, were beaten up.”

The findings show that in particular in the German society, people are excluded or discriminated based on their ethnicity, and their status in society: low social strata, a specific regional area in which Covid-19 is high or their ethnic background. These intersectionality are linked to Covid-19 and shame.

#### Denial and Avoidance and Stigmatization

Denial and avoidance are further connected to Covid-19 and shame and it leads to the neglect of rules and regulations. A Romanian immigrant in South Africa, female, 45-years-old (P24) explained: “In my neighborhood, I noticed that people did not always take the pandemic seriously. They did not respect the rules put in place by the government. Being a foreigner did not allow me to understand their approach in depth. I could only observe them.”

The denial and avoidance of thoughts and actions concerning Covid-19 and its potential consequences and possible counteractions, evokes irresponsible behaviors in society. The missing awareness of Covid-19 and shame leads to risky, non-reflective and non-caring behavior which puts people at risk. This might be connected to the idea that people experience “too little” shame and/or that they do not interlink Covid-19 and shame.

#### Too Little or No Connection Between Covid-19 and Shame

P6, an 82-year-old U.S. male participant pointed out:

“I don't know enough to answer that question, but I think a lot of people in my society feel no shame about possibly infecting others and no shame about the poor or inadequate medical care and income support available to poor people, many African Americans, many Native Americans, many immigrants.”

This participant ascribes irresponsible, risky behavior to people not having or experiencing any shame when potentially spreading the virus to other people. A 68-year-old male British participant (P13) noted:

“You tie Covid-19 and shame together. I don't feel any sense of that at all. This is a personal, and national tragedy. My heart breaks for the impacts and losses here and internationally. Society? I think there was a lot of caution to begin with. I feel people are exhausted and depressed by it now and are therefore not taking care as they might have done before. I believe many decisions being made nationally are now political rather than health related, and many are poor, blind decisions, ignoring the poverty that is worsening in this crisis.”

The participant does not interlink Covid-19 and shame, but rather emotions, such as depression and exhaustion, as well as sadness that he links to the pandemic.

On another note, a 48-year-old Canadian female participant (P8) also declined to connect Covid-19 and shame: “Our local health and political leaders and prime minister never shame others and constantly remind people to be kind to others at this time.”

This statement rather points out the importance of kindness and care to transform the pandemic's challenges. This statement is significant since it highlights a way in which leadership can act in a positive governing way.

Summarizing, the behavior in the context of Covid-19 and shame in various socio-cultural settings and societies are heterogeneous. The connotations tend to be negative in terms of specific groups in society who are excluded, discriminated against, stigmatized, avoided, or denied on one hand, while on the other, no shame or connection between shame and Covid-19 is acknowledged. Negative and positive views on shame in connection with the pandemic seem to be pretty balanced.

## Discussion

The aim of the article was to contribute original, new and in-depth experiences of individuals from different socio-cultural and international backgrounds who share their experiences of shame in the context of Covid-19.

Findings contribute to the discourse that Covid-19 impacts partly negatively on mental health and well-being (Restubog et al., 2020). As described by Bai et al. (2004), people feel stigmatized, ostracized, and rejected during the pandemic. The findings support the idea previously debated that emotional strain needs to be transformed during the pandemic (Restubog et al., 2020; Sun et al., 2020). As described before (Mayer and Vanderheiden, 2019; Mayer et al., 2021), this research study points to the coping mechanisms to deal with shame constructively through different strategies which are influenced by individual and socio-cultural preferences. As shown in this study, participants strive for a meaningful, conscious, and positive life—as highlighted in the PP2.0 literature (Wong, 2011; Passmore and Howell, 2014; Ivtzan et al., 2017; Carreno and Pérez-Escobar, 2019).

This study further supports the idea of Scheff (2020) that shame—at least to a certain degree—impacts on relationships and brings people closer together during Covid-19. This is at least the case in US and European cultures where social support and conversation seems to be one preferred strategy to transform shame during Covid-19. Walsh's (2020) finding that one's own vulnerability during the pandemic can trigger emotions such as shame is strongly supported by this study. The suffering from shame in Chinese contexts, presented by Duan et al. (2020) is also reported in other cultural contexts in this study. In this study, however, participants do not report shaming of governments (as in Vietnam presented by Max, 2020), but rather highlight the shaming of individuals by individuals (as presented for the Taiwanese context by Farr, 2020). Findings further support the research from India where people are ashamed due to infected family members and anxious due to social exclusion (see Sahoo et al., 2020). As described by Kato et al. (2020), also individuals in this study report that they do leave friends and community in the unknown about their status. As further highlighted by Shigemura and Kurosawa (2020), shame and stigmatization go hand in hand even in this study, more with Covid-19 sufferers than with health care providers. They have, in this study, not been named as victims of shame and stigma. Other topics emphasized in the literature, such as stigmatized women (Siddiqui and Nowshin, 2020) or overweight people (Le Brocq et al., 2020) are not mentioned.

Finally, in a way, what is called *jishuku keisatsu* (“self-restraint police”) (Katafuchi et al., 2020) or described for the Swiss context (Nivette et al., 2021), is also to a certain extend mentioned in the study when individuals talk about the positive effect of shame as a mechanism of social control. Shaming is seen as a punishment for those who might not stick to the rules and regulations and therefore an accepted social intervention to ensure social control.

**Figure 3:**
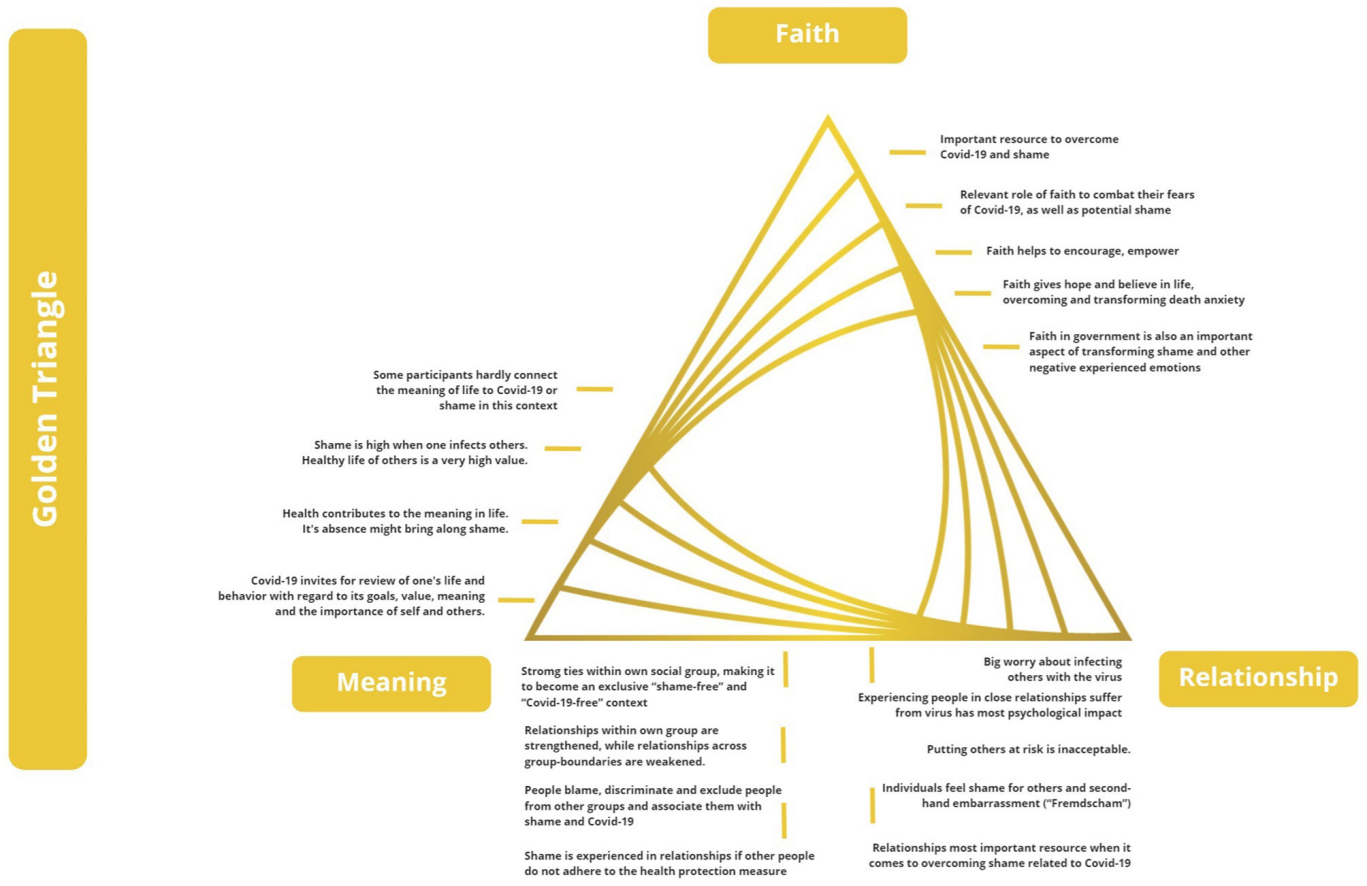
Findings with regard to the Golden Triangle, based on Wong (2019).

**Figure 4:**
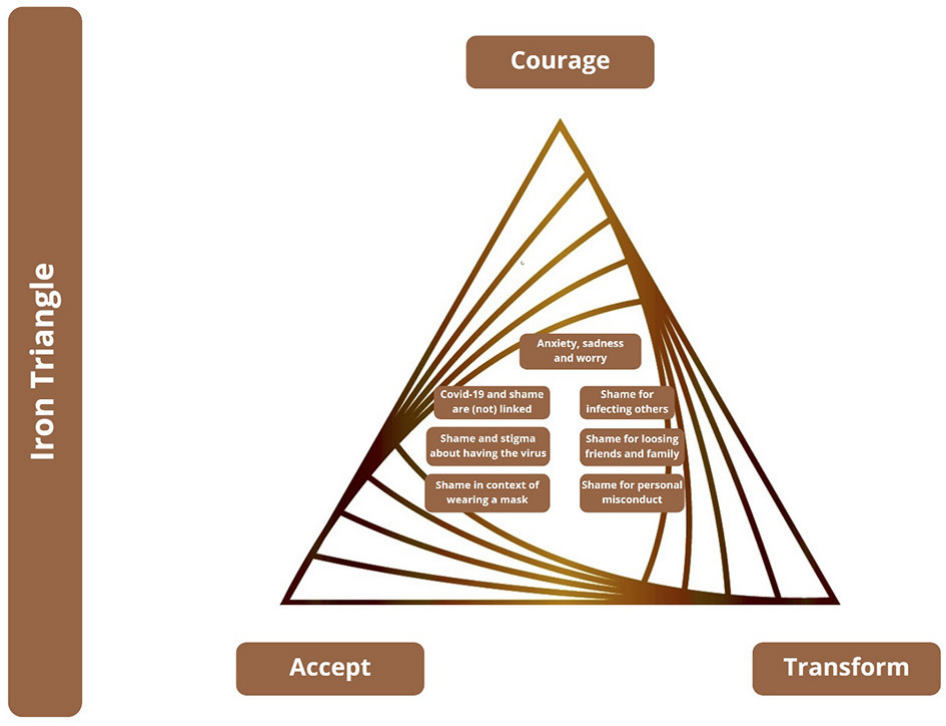
Findings with regard to the Iron Triangle, based on Wong (2019).

The findings are additionally discussed and contextualized with regard to Wong's (2019) “golden triangle” (Figure 3) and the “iron triangle of terror.” Wong (2019) proposes that the golden triangle supports mental health, while the iron triangle (Figure 4) deals with terror and the transformation of negative experiences toward health resources.

### The Golden Triangle of Mental Health

#### Faith (in a Transcendental Reality and Intrinsic Value of Life)

Faith, in a spiritual and/or religious sense, is mentioned as a broadly defined concept and term which focuses mainly on faith in the value of life and as a transcendental reality. It is a resource in the process of overcoming Covid-19 and shame for the participants. The participants mention faith and belief as important resources. However, findings from this research suggest that mainly individuals from the African continent (with African nationality or living in Africa) consider faith as an important part of their transcendental reality to combat their shame caused by Covid-19. Faith also plays a role when transforming potential shame. Faith helps further to encourage, empower and give hope and belief in life, overcoming and transforming death anxiety.

Besides faith as a spiritual value, faith in the government is also an important aspect of transforming shame, anxiety and fear. In this study, participants mention faith (in the sense of trust) in authorities, governments and decision-makers, which helps to transform shame and shaming during the pandemic.

#### Meaning (Life Goal or Value More Important Than Self)

A few participants were shocked and surprised of having contracted the virus. They did not expect to contract the virus. Since the experience increase their death anxiety, they reviewed their life, values and behavior with regard to their goals, meaning, and priorities. A meaningful life is connected to health and well-being and not doing harm to others, not risking their lives due to infecting them with Covid-19. Shame is connected to decreasing meaning in life through unmet goals and values.

Generally, participants hardly referred to the meaning of life during Covid-19 and shame experiences. However, they mention that thinking about life and its value increased during Covid-19.

#### Relationship (Mutual Trust and Care)

A majority of participants explained that they worry most about contracting and infecting others with the virus. Only one elderly man from the U.S. expressed his anger against people who do not care about infecting others. The relationship with others in terms of caring for them and trusting them is highly valued. Putting others at risk is unacceptable and participants suffer most when close family and friends are affected by the virus.

Shame is strong when people do not adhere to the health protection measures and thereby risk the life of others. Individuals feel shamed for others, vicarious embarrassment (“*Fremdscham*”) and hurt, owing to the loss of mutual trust and care.

Relationships are the most important resource in transforming Covid-19 related shame. Social support—built on trust and care—is greatly valued and is primarily experienced through open conversations about the pandemic and associated emotions.

At a societal level, participants recognize that people in society blame, discriminate, and exclude people from other groups and associate them with shame and Covid-19. Meanwhile they strengthen the ties within their own group, creating an imagined and exclusive “shame-free” and “Covid-19-free” context. Relationships within the own group are strengthened, while relationships across group boundaries are weakened.

### The Iron Triangle of Terrors of Life

#### On Courage (to Face the Dark Side of Life)

Most of the participants explained how to face the dark side of Covid-19. Facing the feared frees anxiety, fear, worries, sadness, hurt, depression, and shame. Participants encounter their negative feelings while becoming aware of them. Despair and vulnerability are consciously experienced, but participants are hesitant to face them courageously.

Responding to the question of how shame and Covid-19 are interlinked, one third of the participants did not see any relationship. This might indicate individual and socio-cultural influences affecting how shame is understood. Individuals from Germany, the United Kingdom, and the U.S. could not see any connection of Covid-19 and shame. They might not be courageous enough to face the disease on societal levels.

Participants who are infected, facing the dark side of Covid-19, telling their friends and family about it, experienced rejection, coldness, and aversion. Openness about one's own status needs courage to deal with potential negative reactions.

#### Acceptance (What Cannot Be Avoided or Changed)

The fact of having contracted Covid-19 cannot be avoided or changed. Participants accepted the reality of Covid-19; however, while accepting it, they felt scared, anxious, worried, and concerned. The worry individuals experienced related in particular to their fear of infecting others and thereby becoming responsible for their ill-health.

The findings further reveal many experiences of stigma in connection to shame which call for the transformation from suffering to becoming a “propelling force toward change,” as highlighted, for example, by Participant 24.

Participants showed that shame is experienced when people lose friends or family to Covid-19. Death cannot be avoided or changed and leads to shame. The reality of death anxiety and shame needs to be accepted.

The findings reveal a tendency of non-acceptance of Covid-19 in society. Instead, there is a trend to deny or avoid it and stigmatize “the other”—excluding, and discriminating against members of specific, non-accepted socio-cultural groups. This shows that people hesitate to face the dark side of the disease consciously and actively, but rather project the disease on members of other groups.

#### Transformation (Suffering Into Strength Through Meaning)

With regard to shame and wearing a mask, several participants of different backgrounds highlight that shame is transformed from being a negative feeling toward a health resource when it helps people to comply with health standards, ensuring the health safety of others.

After having accepted the shame and the stigma which comes with contracting Covid-19, the suffering needs to be transformed by making the experience of Covid-19 a meaningful one.

Psychological counseling is one possibility to transform Covid-19 experiences and shame. Professional help is used to transform suffering into strength and meaningfulness.

## Conclusions and Recommendations

The aim to present findings of shame and Covid-19 was followed, presenting new insights on the topic within the framework of PP2.0.

Covid-19 is experienced in connection with negative feelings, such as anxiety, sadness, concern, worry, anger, hopelessness, desperation, depression, and shame.

In conclusion, 1/3 of the participants did not see a connection between Covid-19 and shame while 2/3 see shame when people do not adhere to governmental rules and regulations (e.g., wearing a mask), when contracting or spreading the virus. Spreading the virus is particularly shameful for German female participants, while African participants experience high degree of shame when friends and family lost others to the disease. The phenomenon of feel ashamed for another person's behavior of non-compliance with governmental rules is also discussed in the data.

If participants experience shame in the context of the pandemic, they use different strategies to deal with it, such as increased social support and open discussion (mainly mentioned by European and US participants) as well as spirituality, faith and mindfulness (mainly mentioned by African participants). A very few people use counseling, increase their knowledge or rely on government decisions to transform their negative emotions.

Participants witness people in their societies who try to avoid, deny, exclude, and discriminate against Covid-19 and shame and project shame and fear onto members of marginalized societal groups, such as the socially and economically weak, foreigners, migrants, and immigrants. Obviously the socio-cultural background, age group, and gender of participants influences their thoughts and feelings on the pandemic and shame.

Finally, Covid-19 and the experience of shame in the context of PP2.0 and the triangles of mental health and terror, faith (mainly on the African continent), meaning (hardly referred to in the data), and relationships (very strongly referred to in Europeans and US citizen), impact strongly on transforming shame during the pandemic. In terms of the iron triangle of terrors of life, participants highlight the dark sides of Covid-19 and shame for contracting the disease and infecting others. Generally, participants reflect and aim at accepting the situation and the related shame. However, they seem to experience high levels of untransformed exclusion, denial, and stigmatization when it comes to the pandemic and shame in their societies. The transformation from suffering and pain toward recreating meaning, courage, and acceptance still seems to be in the beginning of the transformational process of individuals across cultures. They do not seem to be courageous enough to suffer to finally transform the negative emotions connected to Covid-19.

Future research in the context of Covid-19 and PP2.0 should apply mixed method research methodologies and explore the process of how shame is transformed at individual, organizational, and socio-cultural levels. Intersectionalities, such as culture, social strata, educational background, first language, gender, age, and the political system should be researched further in terms of their influences.

Professional practitioners, such as counselors, coaches, and therapists need to increase their awareness toward shame impacts during the pandemic. Shame needs to be recognized by professionals as an emotion besides anxiety, depression, sadness, frustration, and isolation since it impacts strongly on many individuals across cultures. With reference to the findings of this study, psychological practitioners need to focus on accepting and transforming suffering and pain caused by the pandemic to increase meaning, strengths, and mental health beyond shame.

## Data Availability Statement

The raw data supporting the conclusions of this article will be made available by the authors, without undue reservation.

## Ethics Statement

The studies involving human participants were reviewed and approved by Viadrina. The patients/participants provided their written informed consent to participate in this study.

## Author Contributions

C-HM and EV have contributed to the article in an equal extent. All authors contributed to the article and approved the submitted version.

## Conflict of Interest

The authors declare that the research was conducted in the absence of any commercial or financial relationships that could be construed as a potential conflict of interest.
